# Effect of Ultrasound Combined With Microbubble Therapy on Interstitial Fluid Pressure and VX2 Tumor Structure in Rabbit

**DOI:** 10.3389/fphar.2019.00716

**Published:** 2019-06-26

**Authors:** Qianyun Zhang, Hai Jin, Liping Chen, Qiaoli Chen, Yan He, Yuwen Yang, Suihong Ma, Shuyi Xiao, Fen Xi, Qiong Luo, Jianhua Liu

**Affiliations:** ^1^Department of Medical Ultrasound, Guangzhou First People’s Hospital, Guangzhou, China; ^2^School of Medicine, South China University of Technology, Guangzhou, China; ^3^Department of Medical Ultrasound, Guangzhou Women and Children’s Medical Center, Guangzhou, China

**Keywords:** blood vessel, collagen fiber, extracellular matrix, interstitial fluid pressure, reticular fiber

## Abstract

Interstitial fluid pressure (IFP) in tumor tissue is significantly higher than that in normal tissue, which reduces the effectiveness of therapeutic drugs. There are several methods to decrease the IFP, such as normalizing blood vessel, decreasing hyaluronic acid and collagen fiber content in the extracellular matrix (ECM), and recovering lymphatic function. Reducing tumor IFP might be developed as a novel approach in cancer therapy. In this study, we aimed to elucidate the relationship between ultrasound combined with microbubble therapy and IFP, and the associated mechanism. VX2 tumor in rabbit was treated with ultrasound combined with microbubbles at different intensities. The IFP was measured using the wick-in-needle (WIN) method. The collagen and reticular fibers were stained by Masson and Gordon–Sweets, respectively. The results showed that low-frequency non-focus ultrasound combined with microbubbles therapy influences the IFP in tumor tissues; low-frequency non-focus ultrasound with low pressure increased the IFP, whereas middle–high pressure decreased the IFP. The results showed that the structure and content of collagen and reticular fibers in tumor tissue were rarely influenced by the treatment. Our study provides a novel approach of reduced IFP antitumor therapy.

## Introduction

Increasing incidence of cancer and associated mortality have forced innovations in tumor therapy. It has been reported that higher interstitial fluid pressure (IFP) in solid tumors leads to lower penetration efficiency of chemotherapy drugs from the capillary to tumor tissues, consequently limiting their antitumor effect (Heldin et al., 2004).

IFP is determined by hydrostatic pressure and oncotic pressure in the capillary and interstitial space, and it is also influenced by hydraulic conductivity and plasma protein reflectance. The pressure in normal tissues is slightly negative, ensuring easy material penetration from the blood vessels to the interstitial space. On the contrary, the pressure in many solid tumors is positive.

The tumor IFP is increased by several factors, such as abnormal blood vessels (Chen and Shi, 2002), dense extracellular matrix (ECM), abnormal fibrosis (Dufort et al., 2016), and abnormal lymphatic vessel (DiResta et al., 2000; Padera et al., 2004; Alitalo et al., 2006; Hagendoorn et al., 2006). Reducing tumor IFP might be developed as a novel approach in antitumor therapy. There are several methods to decrease the IFP, such as normalizing blood vessel (Lee et al., 2000; Tong et al., 2004; Willett et al., 2004; Goel et al., 2011), decreasing hyaluronic acid and collagen fiber content in the ECM (Brekken and de Lange Davies, 1998; Brekken et al., 2000a; Brekken et al., 2000b), and recovering lymphatic function (Starling; Young et al., 1950; Jain, 1987a; Jain, 1987b).

The ECM in animals, including the interstitial matrix, basement membrane, polysaccharide gel, and fibrin, consists of the interstitial matrix that fills the intercellular space to buffer various external stresses. Tumor ECM, including mesenchymal cells (fibroblasts, astrocytes, and inflammatory cells), collagen fibrils, glycosaminoglycans, and proteoglycans, which act as scaffolds to support growing tumor cells, separate tumor cells from blood vessels to increase the IFP and to compress tumor blood vessels and lymphatic vessels, resulting in lower blood flow and even collapse blood vessels (Jain, 1987a; Jain, 1987b; Less et al., 1992; Nathanson and Nelson, 1994). However, systematic studies on the ECM are limited owing to the complexity of its components.

Applying microbubbles in ultrasonic therapy is a hot spot. High-intensity ultrasound combined with microbubble (US-MB) therapy blocked blood flow to transplanted tumor in mice for 24 h and low-intensity US-MB therapy increased tumor perfusion temporarily (Matsumura and Maeda, 1986). However, there is no systematic research to clarify the relationship between non-focused US-MB therapy and tumor IFP. The aim of this study was to identify a novel approach in antitumor therapy by clarifying the relationship between US-MB therapy and tumor IFP, and the associated mechanism. Therefore, we established a rabbit model with VX2 tumor. The tumors were treated with different intensities of US-MB.

## Materials and Methods

### Animal Model

Healthy New Zealand white rabbits weighing approximately 2.5 kg were obtained from an experiment center in Guangdong, China. Before inoculation, all rabbits were reared for at least 7 days at 24°C–26°C under 45–55% humidity. This study was carried out in accordance with the principles of the Basel Declaration and recommendations of *Guide for the Care and Use of Laboratory Animals* published by the United States National Institutes of Health (NIH publication no. 85-23, revised 1996). The protocol was approved by the Laboratory Animal Committee (LAC) of South China University of Technology, Guangdong, China.

VX2 tumor tissue specimens were obtained from the cell bank of Sun Yat-sen University (Guangzhou, China). The tumor tissues were chopped into small pieces (1 mm^3^) and placed in a culture dish with physiological saline solution, and then injected subcutaneously to the superficial muscles of the left hind limb of rabbits. The experiment was performed for almost 10 days after tumor implantation, until the tumor reached a size of approximately L (length) 10 ± 0.7 mm and W (width) 5 ± 0.8 mm.

### Experimental Procedure

**Figure 1:**
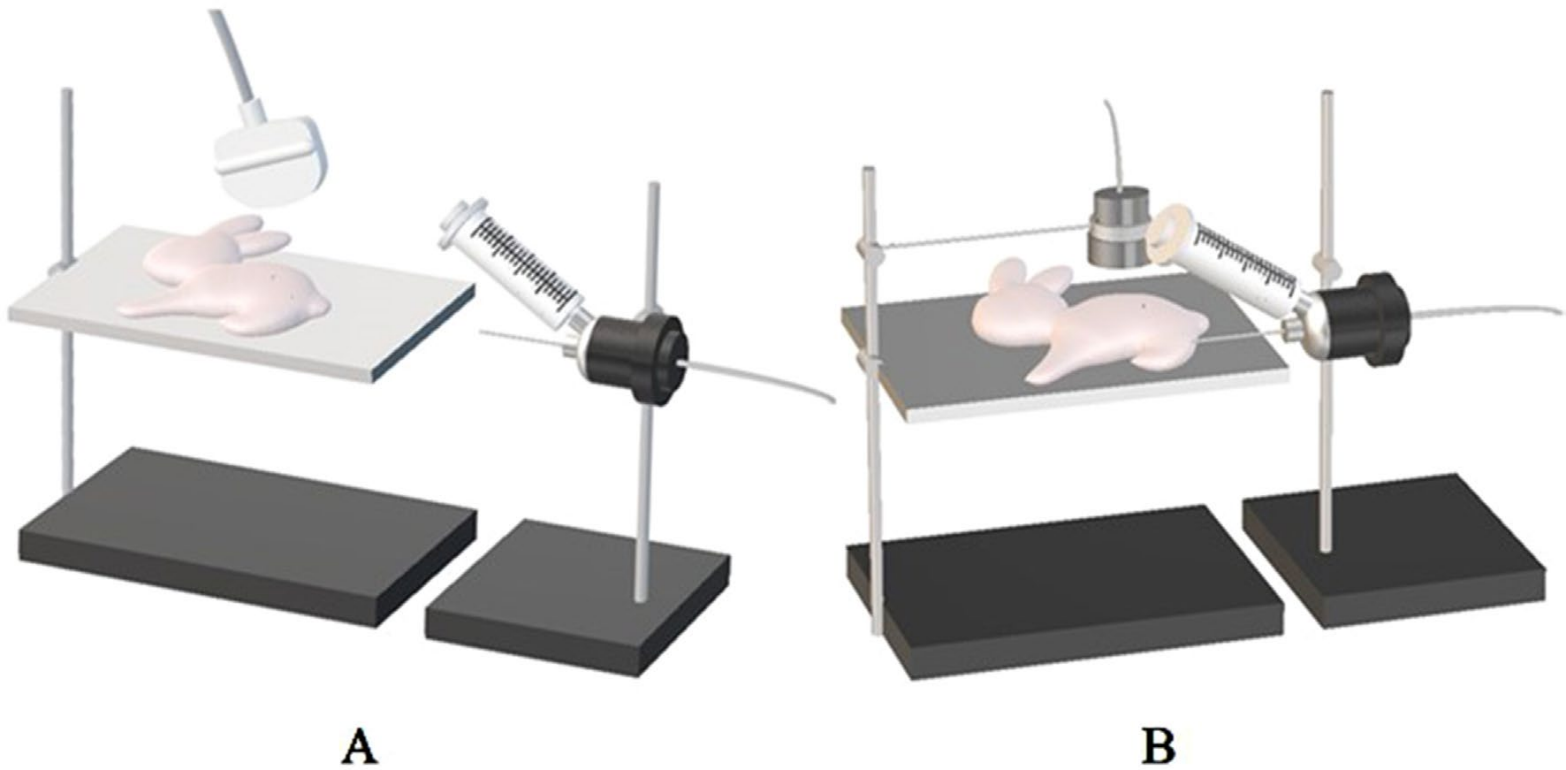
**(A)** The puncture needle is connected to the bio-signal acquisition and analysis system and placed horizontally at the same level as the tumor. The needle was inserted horizontally into the center of the tumor under ultrasound guidance, and the stable reading as the corresponding interstitial fluid pressure (IFP) value. **(B)** The therapeutic probe of ultrasonic cavitation therapeutic instrument is fixed on the iron frame, and the therapeutic probe is placed on the corresponding body surface of the tumor with sufficient coupling, and the tumor is irradiated with energy of 1 MPa, 3 MPa, and 5 MPa according to the set treatment parameters in each group, respectively.

All 48 tumor-bearing rabbits were divided into six groups randomly (*n* = 8 per group). These six groups included the low-intensity ultrasound combined with microbubble (1MPa-USMB), medium-intensity ultrasound combined with microbubble (3MPa-USMB), high-intensity ultrasound combined with microbubble (5MPa-USMB), medium-intensity ultrasound without microbubble (US), microbubble (MB), and blank control (BC) groups. Tumor tissues were examined by contrast-enhanced ultrasound (CEUS) before and after treatment (GE E9, Probe: ML6-15), and the peak intensity (PI) was recorded. The tumor IFP and surrounding tissue IFP were measured using the wick-in-needle (WIN) method. The tumor surrounding tissue IFP was measured before treatment, and the tumor IFP was measured before, during, and after the treatment (**Figure 1A**). After CEUS and measurement of IFP in tumor tissues and surrounding tissues, each group was treated according to the corresponding conditions. In the USMB group, the tumor-bearing rabbits were intravenously injected with 0.1 ml/kg of diluted lipid microbubbles (ZHIFUXIAN, Department of Ultrasound, Xinqiao Hospital Affiliated to Third Military Medical University, Chongqing, China), and the tumor was disposed by low-frequency, non-focused ultrasound of different intensities (acoustic pressure of 1, 3, and 5 MPa) for 5 min, pulse repetition frequency of 10 Hz, and duty ratio of 0.2%. The pulse emission/gap time was 9 s/3 s (**Figure 1B**). In the US group, the rabbits were intravenously injected with the same volume of sterile saline solution, and the tumor was disposed by 3 MPa ultrasound for 5 min. In the MB group, the rabbits were intravenously injected with the same volume of diluted lipid microbubbles, and the tumor was disposed by sham ultrasound exposure for 5 min. In the BC group, the rabbits were intravenously injected with the same volume of sterile saline solution, and the tumor was disposed by sham ultrasound exposure for 5 min. After measuring the tumor IFP, the rabbits were sacrificed. The tumor tissue was removed and fixed with 4% paraformaldehyde solution for 24 h, and then hematoxylin and eosin (H&E) staining, Masson staining, and Gordon–Sweets reticular fiber staining were performed.

### Ultrasound Treatment

Ultrasound treatment was performed using a pulsed therapeutic ultrasound device equipped with a KHT-017 transducer (DCT-700; Shenzhen Well.D Medical Electronic, Shenzhen, China). To maintain a gap of 2 cm between the transducer and the skin, the transducer was fixed to a steel stand with a scale. Subsequently, ultrasound coupling gel was applied to the skin. Treatment was implemented for approximately 5 min. The US-MB treatment was applied to the tumor after intravenous injection of microbubbles at a dose of 0.1 mL/kg. The transducer was operated at a frequency of 1 MHz; an acoustic pressure of 1, 3, and 5 MPa; a pulse repetition frequency of 10 Hz; and a duty cycle of 0.2%. The treatment was performed under an intermittent mode of 9 s on and 3 s off for 5 min.

### Tumor IFP

**Figure 2:**
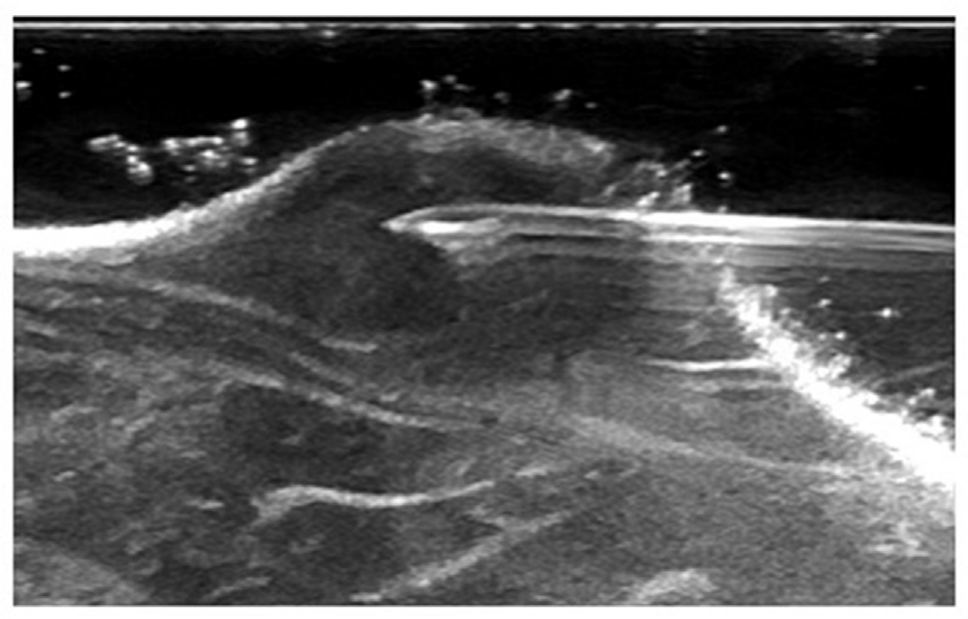
The needle was inserted horizontally into the center of the tumor under ultrasound guidance.

The IFP of the tumor center was measured by the WIN method using a 25-G needle (**Figure 2**).

### H&E Staining

To assess the therapeutic effect of treatments on tumor in each group, the tumor tissue was fixed in formalin, embedded in paraffin, sectioned serially, and stained using H&E. Ten fields of vision were selected randomly and observed using an optical microscope (Axio Scope A1; Zeiss, Oberkochen, Germany). The tumor cells, tumor micro-vessels, and the changes around them were observed by high-power optical microscopy.

### Masson Staining and Gordon–Sweets Reticular Fiber Staining

To assess the effect of treatments on collagen and reticular fibers in each group, the tumor sections were subjected to Masson staining and Gordon–Sweets reticular fiber staining. Ten fields of vision were selected randomly and observed using an optical microscope (Axio Scope A1). Image-pro Plus 6.0 software was used to calculate the content of collagen and reticular fibers in each pathological section.

### Statistical Analyses

Multiple comparisons were performed using the analysis of variance. Paired-sample *t* test was used to compare the differences before and after treatment in each group. The data are expressed as mean ± SD, and the results with *P* value < 0.05 were considered statistically significant. Statistical analyses were performed using SAS 9.4 (SAS Institute Inc. Cary, NC).

## Results

### Contrast-Enhanced Ultrasound

**Figure 3:**
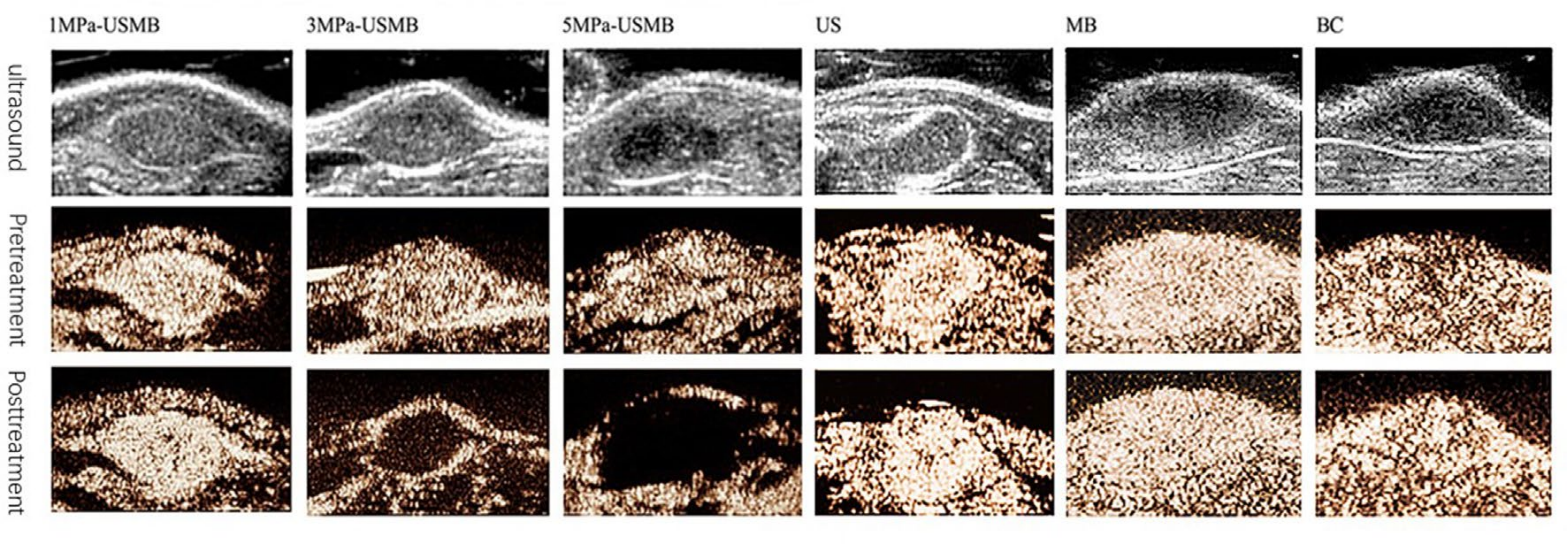
Microbubbles were filled into the tumor rapidly and evenly, and no filling defect was found in all groups before treatment. After treatment, enhancement of the tumor was marginally stronger than pretreatment in low-intensity ultrasound combined with microbubble (1MPa-USMB) group, but in medium-intensity ultrasound combined with microbubble (3MPa-USMB) group, the contrast-enhanced ultrasound (CEUS) showed a filling defect in the center of the tumor with a ring-shaped enhancement around the tumor, and the filling defect was larger in high-intensity ultrasound combined with microbubble (5MPa-USMB) group than in 3MPa-USMB group. The blood perfusion after treatment in US group, MB group, and BC group were similar, and the microbubbles were filled into the tumor quickly and completely.

Contrast-enhanced ultrasound (CEUS) was performed before and immediately after treatment in all the groups. Microbubbles filled into the tumor rapidly and evenly, and no filling defect was observed in all the groups before treatment. After treatment, enhancement of the tumor was marginally stronger than that before treatment in the 1MPa-USMB group, but in the 3MPa-USMB group, the CEUS showed filling defect in the center of the tumor with a ring-shaped enhancement around the tumor. Furthermore, the filling defect was larger in the 5MPa-USMB group than in the 3MPa-USMB group. Blood perfusion after treatment in the US, MB, and BC groups was similar, and the microbubbles filled the tumor quickly and completely (**Figure 3**).

**Figure 4:**
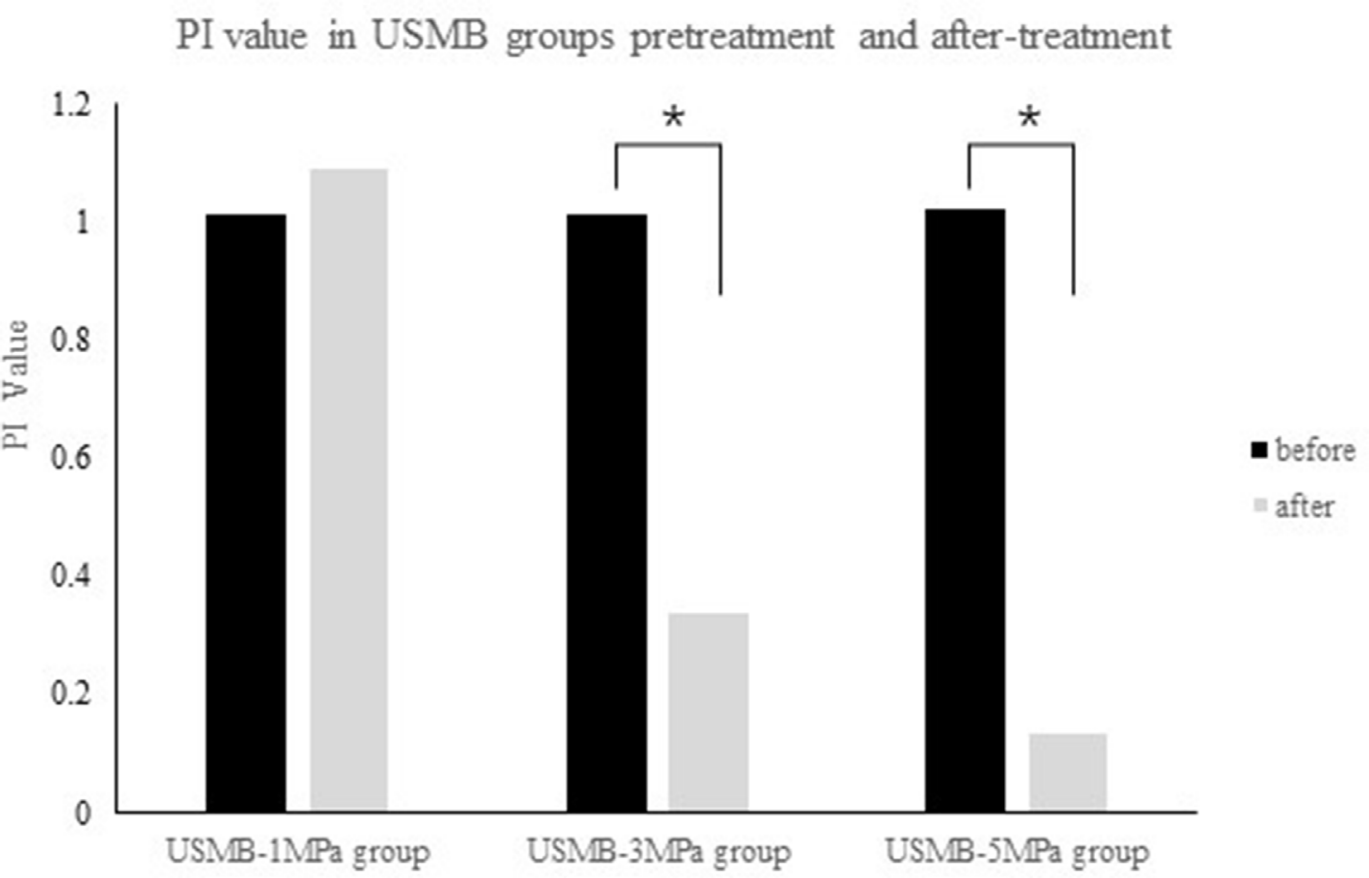
The not significantly different in peak intensity (PI) was not significantly different in 1MPa-USMB group pre- and posttreatment. The PI value in 3MPa-USMB group and 5MPa-USMB group decreased 66.3% and 86.7% (*p* < 0.05), respectively, after treatment.

The PI, which indicates blood perfusion in tumor tissues, was recorded and analyzed. The pretreatment PI was not significantly different among the groups. The post-treatment PI was not significantly different in the US, MB, BC, and 1MPa-USMB groups. The PI in the 3MPa-USMB and 5MPa-USMB groups decreased by 66.3% and 86.7% (*p* < 0.05), respectively, after treatment compared with that before treatment. The results are shown in **Figure 4**.

### Interstitial-Fluid Pressure

**Figure 5:**
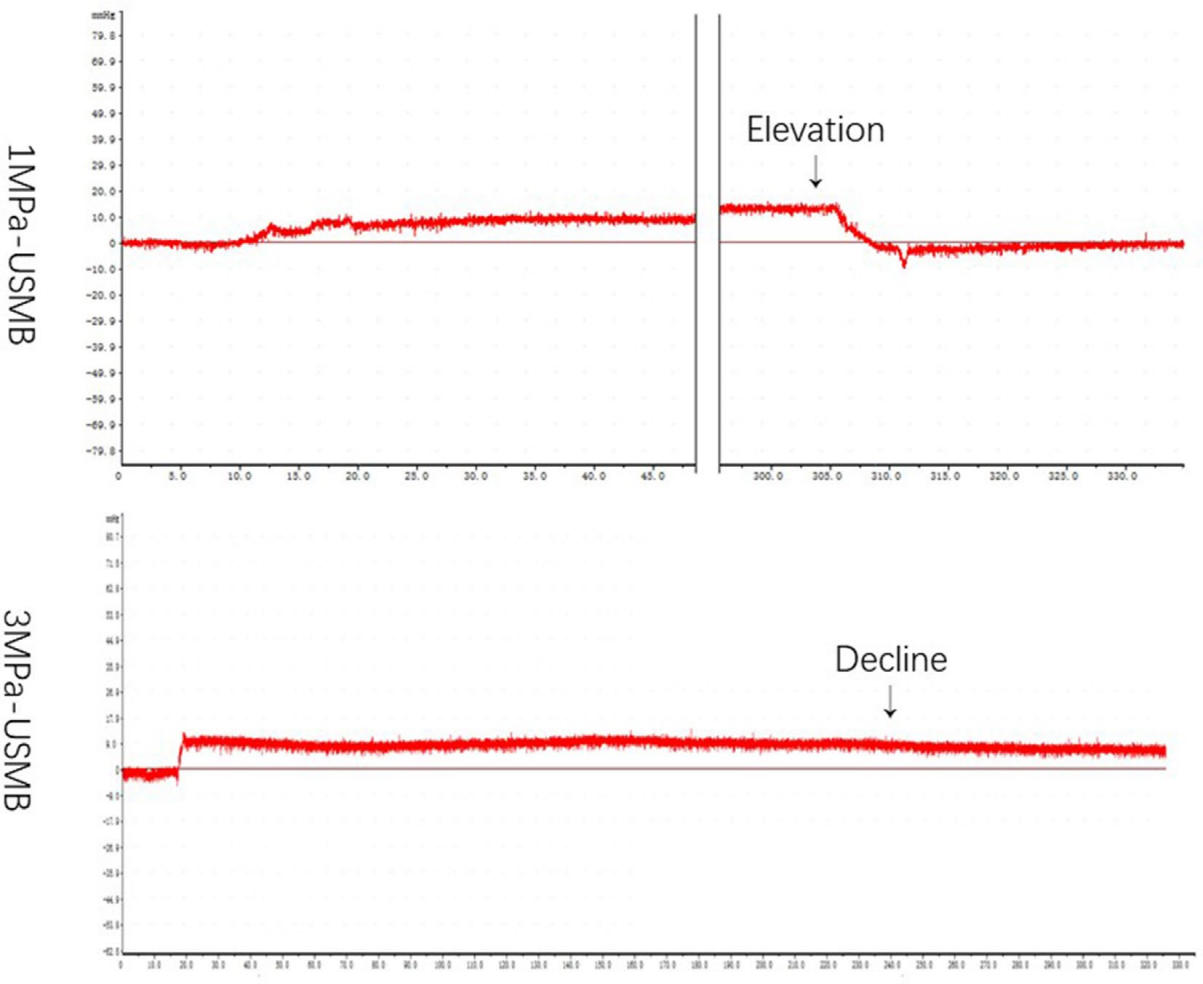
The curve of IFP level in USMB group post-treatment: in 3MPa-USMB group and 5MPa-USMB group, the curve decreased steadily during the treatment, while it increased steadily in 1MPa-USMB group.

**Table 1** summarizes the IFP in each group before and after treatment. The overall mean IFP was 14.5 ± 6.8 mmHg in the tumor tissues and −6.7 **±** 3.2 mmHg in the peripheral tissues. **Figure 5** shows the curve of IFP in the USMB groups post treatment. In the 3MPa-USMB and 5MPa-USMB groups, the curve decreased steadily during treatment, but it increased steadily in the 1MPa-USMB group. The IFP showed no significant change in the BC, US, and MB groups during the treatment. After treatment, the tumor IFP in the 1MPa-USMB group was slightly higher than that before treatment (*p* < 0.0001), but lower than that in the 3MPa-USMB and 5MPa-USMB groups (*p* < 0.0001). However, there was no significant difference in the IFP change between the 3MPa-USMB and 5MPa-USMB groups (*p* > 0.05).

**Table 1:** The interstitial fluid pressure (IFP) level in each group preprocedural and postprocedural (mmHg).

	BC	US	MB	1MPa-USMB	3MPa-USMB	5MPa-USMB
Preprocedural Postprocedural	13.2 ± 9.7 13.2 ± 9.5	11.8 ± 6.1 11.7 ± 6.5	18.0 ± 6.9 18.1 ± 7.0	11.0 ± 3.3 13.0 ± 3.4	17.7 ± 4.9 12.9 ± 5.5	15.3 ± 7.3 9.8 ± 7.6
*p*				*	*	*

*p < 0.0001, preprocedural versus postprocedural.

The range of IFP level in tumors: 10–40 mmHg.

### H&E Staining

**Figure 6:**
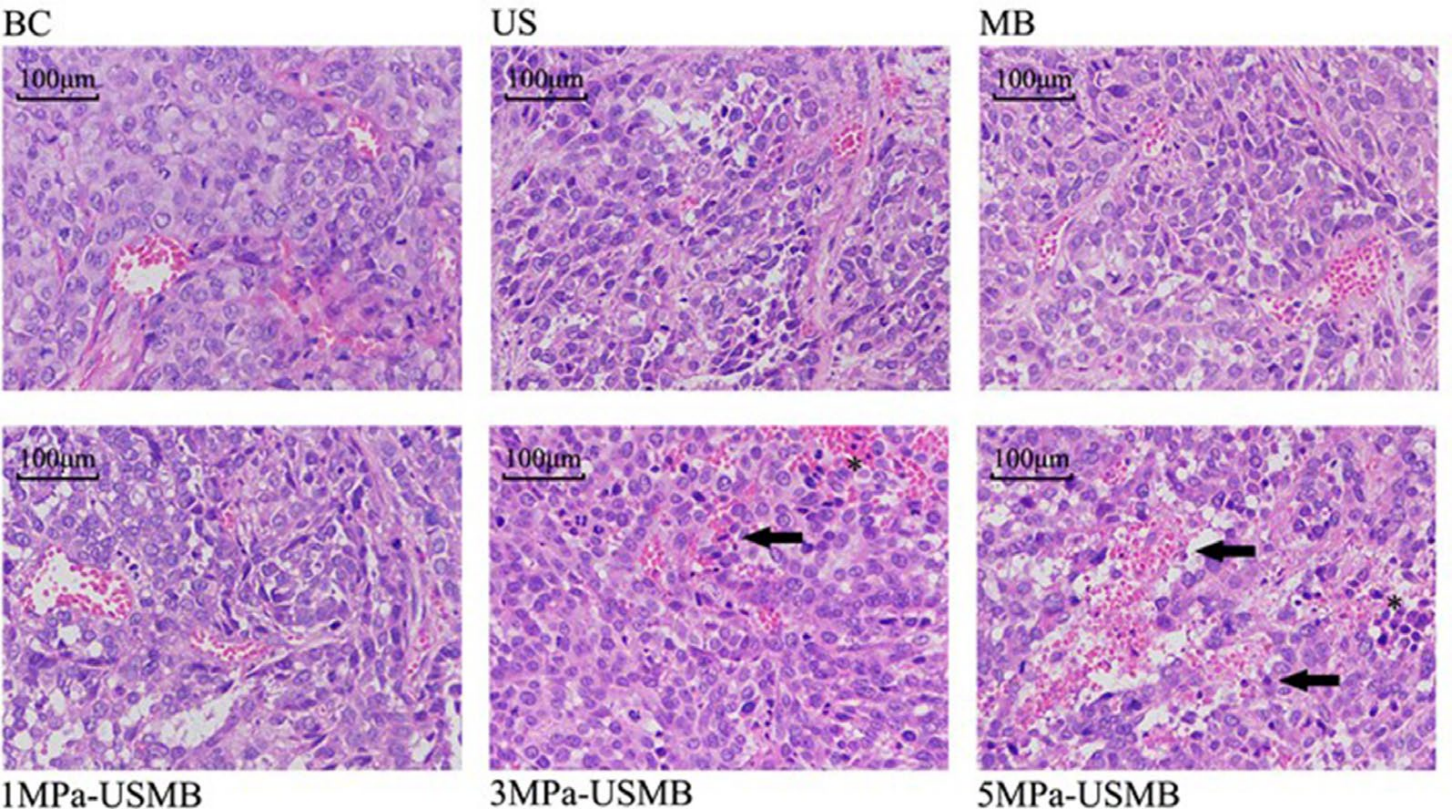
The tumor cells are disordered, different in size, densely arranged, large nuclei stain and nuclei dimorphism. The tumor blood vessels were branched and their structure was clear. Furthermore, the vessel wall was intact and continuous, with no obvious damage. There are no red blood cells observed at the periphery of the vessels in US group, MB group, and BC group. There are some red blood cells observed at the periphery of the vessels in 1MPa-USMB group. The microvascular congestion and expansion can be seen in the injured area, the vessel-wall is incomplete (solid arrow), and red blood cells can be seen around the vessels (asterisk) in 3MPa-USMB group and 5MPa-USMB group.

The pathological results were similar among the US, MB, and BC groups; the tumor cells were disordered, varied in size, and densely arranged, with large and dimorphic nuclei. The tumor blood vessels were branched and their structure was clear. Furthermore, the vessel wall was intact and continuous, with no obvious damage. Red blood cells were not observed at the periphery of the vessels. H&E staining revealed that the tumor from the 1MPa-USMB group was similar to that from the US, MB, and BC groups, but there were some red blood cells at the periphery of the blood vessels. Focal necrosis of tumor cells was found in the 3MPa-USMB and 5MPa-USMB groups. Microvascular congestion and expansion were observed in the injured area, and the vessel wall was incomplete and red blood cells were observed around the vessels, which was more obvious in the 5MPa-USMB group than in the 3MPa-USMB group (**Figure 6**).

### Masson Staining and Gordon–Sweets Staining

**Figure 7:**
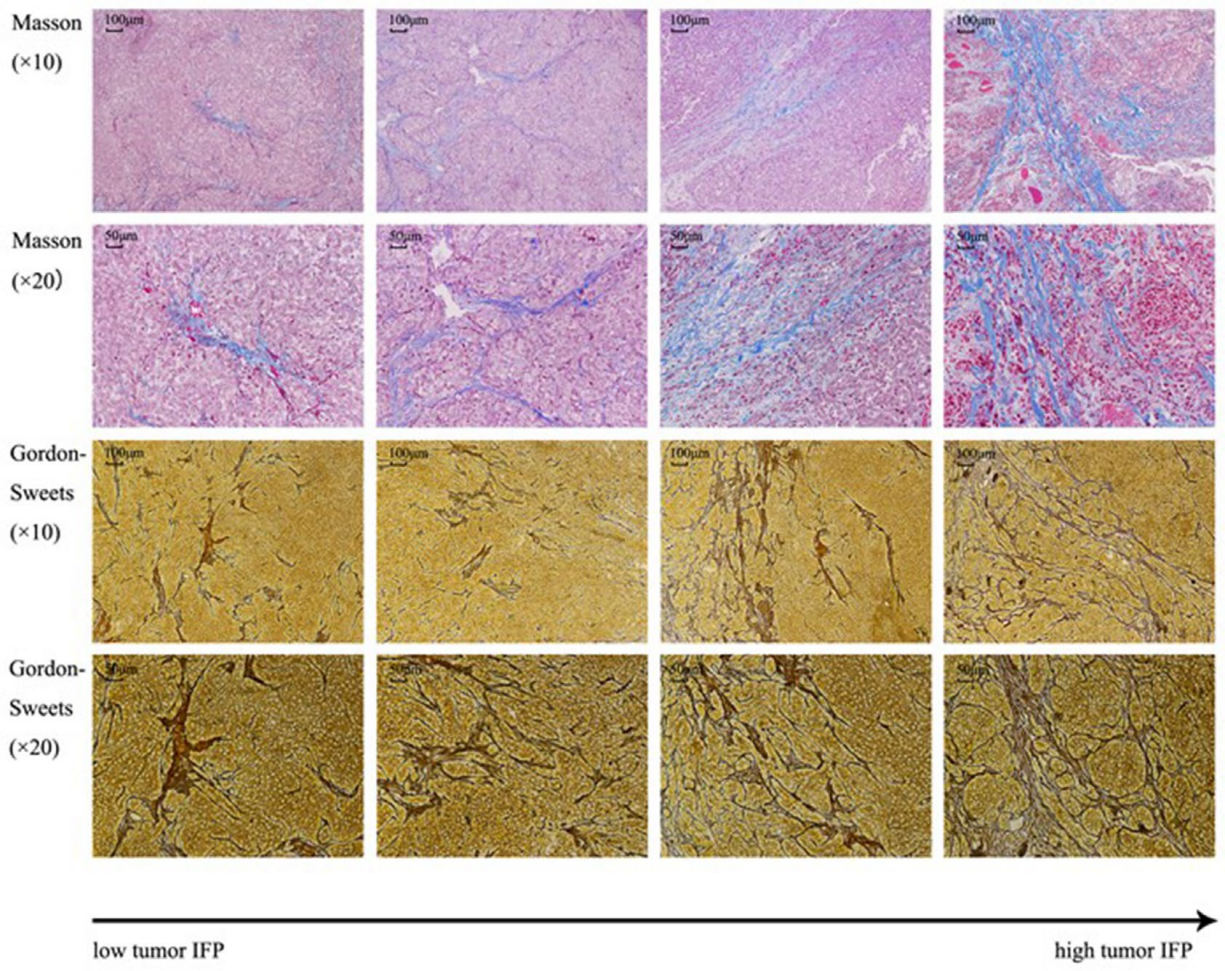
Masson staining and Gordon–Sweets staining. In Masson staining, collagen fibers were indicated in blue, cytoplasm was indicated in red, and nucleus was indicated in blue. In Gordon–Sweets staining, reticular fibers were indicated in brownish black.

**Figure 8:**
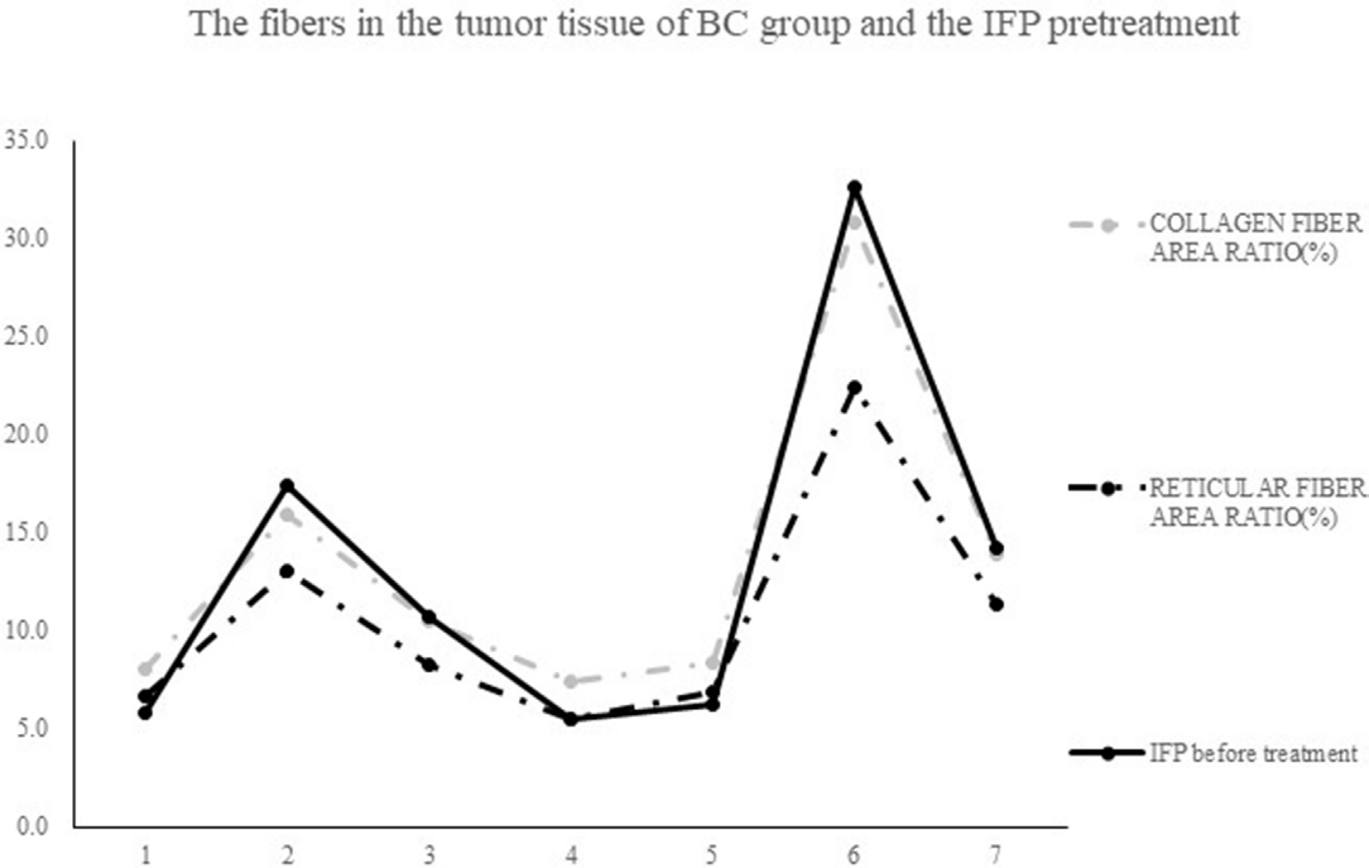
The contents of collagen fibers and reticular fibers in BC group correlation with the level of pretreatment tumor IFP: the higher the contents of collagen fiber and reticular fiber, the higher the IFP level.

**Figure 7** shows the tissue sections subjected to Masson staining and Gordon–Sweets staining. In the Masson-stained sections, the collagen fibers appeared blue, cytoplasm appeared red, and nucleus appeared blue. In Gordon–Sweets-stained sections, the reticular fibers appeared brownish black. The BC group represented the pretreatment state of the tumor tissues. **Figure 8** shows that the content of collagen fibers and reticular fibers in the BC group correlated with the pretreatment tumor IFP; the higher the content of collagen fiber and reticular fiber, the higher the IFP. **Table 2** summarizes the ratio of collagen and reticular fibers in each group. The results showed that there was no significant difference between each group (*p* = 0.27 and *p* = 0.14). This indicated that our treatment has little or no effect on the content of collagen and reticular fibers in tumor tissues.

**Table 2:** The ratio of collagen and reticular fibers in each group.

	BC	US	MB	1MPa-USMB	3MPa-USMB	5MPa-USMB
Collagen fibers (%)	13.6 ± 8.2	11.8 ± 4.0	16.8 ± 5.9	11.4 ± 2.6	16.8 ± 5.1	14.7 ± 7.0
Reticular fibers (%)	10.6 ± 5.9	8.9 ± 3.6	13.6 ± 5.0	9.2 ± 2.3	14.0 ± 4.4	11.4 ± 5.3

## Discussion

CEUS can be used to evaluate blood perfusion into tumor tissues. Blood perfusion indicated the IFP in tumor tissues. In the present study, we adopted three different acoustic pressure (1, 3, and 5 MPa) low-frequency ultrasound treatments to represent low-intensity, medium-intensity, and high-intensity ultrasound treatments combined with microbubbles to determine the changes in tumor IFP. There was no significant difference in the CEUS before and after treatment in the 1MPa-USMB group. Studies have shown that above 1MPa-USMB treatment can improve vascular permeability; however, it is based on normal tissues. Vessels in tumor are abnormal and easily influenced by cavitation effect (Heldin et al., 2004; Li et al., 2011). The pathological results showed that the tumor vessels were still clear and intact, and that there was no obvious defect in the vessel wall; leakage of red blood cells was observed around the vessels. We suggest that 1MPa-USMB treatment can improve tumor vascular permeability, allowing more vascular contents to penetrate through the vessel wall into the interstitial space; thus, increasing colloidal osmotic pressure. Therefore, it is reasonable to consider that this mechanism increases the tumor IFP. The post-treatment CEUS in the 3MPa-USMB and 5MPa-USMB groups showed filling defect in the central region of tumor, and the PI decreased significantly. Filling defect indicates blockage in tumor blood perfusion, which is thought to be caused by microvascular destruction in the injured area of tumor tissue. Vessel wall damage, endothelial cell injury, and micro thrombosis are the factors that affect blood perfusion to tumor tissue (Heldin et al., 2004; Li et al., 2011). Studies have shown that vascular disrupting agents (VDAs) decrease the IFP in tumor tissues effectively (Gaya and Rustin, 2005). Based on this finding, it is reasonable to conclude that 3MPa-USMB and 5MPa-USMB treatments decrease the IFP by blocking blood perfusion like VDAs do.

In our previous study, the micro vessel density in tumor tissues was significantly reduced after 3MPa-USMB and 5MPa-USMB treatments, but there was no significant difference between these two treatments. This explains why there was no significant difference in the IFP change between the 3MPa-USMB and 5MPa-USMB groups. The IFP change in the US, MB, and BC groups was not significantly different compared with that before treatment.

Abnormal interstitial structure in tumor tissues increases the IFP, and the collagen and reticular fibers are the scaffold for growing tumor cells in a suitable microenvironment. In the present study, the collagen fibers were stained by Masson staining and the reticular fibers were stained by Gordon–Sweets staining (Lee et al., 2017). The distribution of collagen and reticular fibers in tumor tissue was observed post treatment in each group. As there is no chance to observe these fibers pre treatment, we considered the BC group as the pre-treatment state. Analysis using image analysis software showed that there was no significant difference in the ratio and morphology of collagen and reticular fibers pre and post treatment in each group. That is, the content and structure of collagen and reticular fibers in each group did not change significantly pre and post treatment. The decrease in tumor IFP without changes in the collagen and reticular fibers indicate that the tumor IFP decrease was not due to a reduction in content of these fibers. Previous studies have shown that High-Intensity Focused Ultrasound (HIFU) reduces the tumor IFP by destroying the fibers, especially collagen fibers, due to the thermal and mechanical effects of HIFU (Matsumura and Maeda, 1986; Gaya and Rustin, 2005; Watson et al., 2012; Lee et al., 2017). In our study, we proved that the low-frequency non-focus US-MB has negligible effect on the ECM. Some studies have shown that the degradation of collagen fibers and hyaluronic acid in the matrix reduced the IFP effectively (Ross et al., 2002; Gaya and Rustin, 2005; Okada et al., 2005; Fukumura and Jain, 2007; Qiao et al., 2013; Lee et al., 2017). However, both collagen fibers and hyaluronic acid inhibit tumor growth and metastasis (Itano et al., 2002; Jojovic et al., 2002; Kovar et al., 2006); hence, whether the destruction of collagen fibers and hyaluronic acid in the ECM is beneficial in inhibiting tumor growth by decreasing tumor IFP should be evaluated further. Moreover, studies should be conducted on how to reduce the tumor IFP without breaking this barrier. 

In conclusion, our study results revealed that there is a correlation between the IFP and the fibers in the BC group; the higher the IFP level, the more the collagen and reticular fiber content. This result is consistent with that of previous studies (Starling; Montesano and Orci, 1988; Clark et al., 1989; Boucher et al., 1990; Gullberg et al., 1990; Kitadai et al., 2001; Padera et al., 2004; Fukumura and Jain, 2007). The mechanism involved in the increase in IFP is the fibers restrict tumor deformation, and the mechanical pressure can destroy the lymphatic function (Sheikov et al., 2004; Thakkar et al., 2013; De Cock et al., 2015).

## Conclusions

Low-frequency non-focus US-MB therapy can change the IFP of tumor tissue and cause no significant changes in the structure and content of collagen and reticular fibers in the tumor ECM. Low-frequency non-focus ultrasound with low acoustic pressure (1 MPa) increased the tumor IFP, whereas low-frequency non-focus ultrasound with medium-high acoustic pressure decreased the tumor IFP. There was no significant difference between the medium and high acoustic pressure groups in terms of decrease in the tumor IFP. Our study presents a novel approach of reduced IFP antitumor therapy.

## Author Contributions

QZ, HJ, and JL conceived and designed the experiments and wrote the paper; QZ and HJ performed the experiments and analyzed the data; PC, QC, FX, SX, SM, YY, HY, and QL contributed reagents/materials/analysis tools. All authors provided their approval for publication.

## Funding

This study was supported by the Natural Science Foundation of Guangdong Province (grant no. 2016A030313461).

## Conflict of Interest of Statement

The authors declare that the research was conducted in the absence of any commercial or financial relationships that could be construed as a potential conflict of interest.

## Abbreviations

BC, blank control; CEUS, contrast-enhanced ultrasound; MB, microbubble; MVD, micro vessel density; PI, perfusion index; US-MB, ultrasound combined with microbubble; VDA, vascular disrupting agent.
